# Erratum for Quinn et al., “Bridging the Gap between Analytical and Microbial Sciences in Microbiome Research”

**DOI:** 10.1128/mSystems.01171-21

**Published:** 2021-10-05

**Authors:** Robert A. Quinn, Kehau A. Hagiwara, Ken Liu, Maryam Goudarzi, Wimal Pathmasiri, Lloyd W. Sumner, Thomas O. Metz

**Affiliations:** a Department of Biochemistry and Molecular Biology, Michigan State University, East Lansing, Michigan, USA; b Chemical Sciences Division, National Institute of Standards and Technology, Hollings Marine Laboratory, Charleston, South Carolina, USA; c Clinical Biomarkers Laboratory, Emory University, Atlanta, Georgia, USA; d Cleveland Clinic Lerner Research Institute, Cleveland, Ohio, USA; e Nutrition Research Institute, University of North Carolina at Chapel Hill, Kannapolis, North Carolina, USA; f Department of Biochemistry, Bond Life Sciences Center, MU Metabolomics Center, University of Missouri, Columbia, Missouri, USA; g Biological Sciences Division, Pacific Northwest National Laboratory, Richland, Washington, USA

## ERRATUM

Volume 6, no. 5, e00585-21, 2021, https://doi.org/10.1128/mSystems.00585-21. Page 2: In the Fig. 1 legend, line 11, “reverse transcription [RT]” should read “retention time [RT].”

